# QTL‐Based Evidence of Population Genetic Divergence in Male Territorial Aggressiveness of the Japanese Freshwater Threespine Stickleback

**DOI:** 10.1002/ece3.70795

**Published:** 2025-01-09

**Authors:** Haruka Yamazaki, Seiichi Mori, Osamu Kishida, Atsushi J. Nagano, Tomoyuki Kokita

**Affiliations:** ^1^ Faculty of Agriculture Kyushu University Fukuoka Japan; ^2^ The Institute of Regional Development Gifu Kyoritsu University Ogaki Japan; ^3^ Tomakomai Experimental Forest, Field Science Center for Northern Biosphere Hokkaido University Tomakomai Japan; ^4^ Faculty of Agriculture Ryukoku University Otsu Japan; ^5^ Institute for Advanced Biosciences Keio University Tsuruoka Japan

**Keywords:** aggression, behavioral variation, common‐garden experiment, population divergence, QTL mapping, territoriality

## Abstract

Territorial aggression is widespread across the animal kingdom and is expressed in diverse ecological and social contexts. In addition, there are marked variations in the degree of male reproductive territoriality within and between species. These differences are often attributed to genetic components. However, the evolutionary genetic mechanisms in wild animals are poorly understood. This study explored the genetic basis of divergent male territorial aggressiveness between two Japanese freshwater populations, Gifu (GF) and Tomakomai (TM), in the threespine stickleback, which is a well‐known model system for both behavioral ecology and evolutionary genetics. First, our field survey indicated that the distribution of reproductive territories differed greatly across breeding habitats between the focal populations, and the density of reproductive territories was much greater in the GF population. Second, a one‐on‐one arena aquarium experiment on male–male combat using wild‐caught and common‐garden‐reared males revealed that GF males were genetically more aggressive than TM males. Finally, we performed quantitative trait loci (QTL) analysis using an F_2_ hybrid cross between the two populations to identify the causal genomic regions contributing to the divergence in male territorial aggressiveness. Our QTL analysis identified a single significant locus in an aggression‐related behavioral component, that is, the number of bites of focal F_2_ males toward a GF stimulus intruder. Two notable behavior‐related genes, *HTR2A* and *MAO‐A*, are found near this locus. These genes have often been suggested to influence of aggressive behavior in animals; therefore, they are regarded as important candidate genes for further functional analyses. Thus, we are the first to provide a QTL‐based genetic basis for population divergence in male territorial aggressiveness in the threespine stickleback.

## Graphical abstract

Territorial aggression is widespread across the animal kingdom and is expressed in diverse ecological and social contexts. This study explored the genetic components of divergent male territorial aggressiveness between two Japanese freshwater populations in the threespine stickleback, which is a well‐known model system for both behavioral ecology and evolutionary genetics. We are the first to provide a QTL‐based genetic basis for population divergence in male territorial aggressiveness in this species.

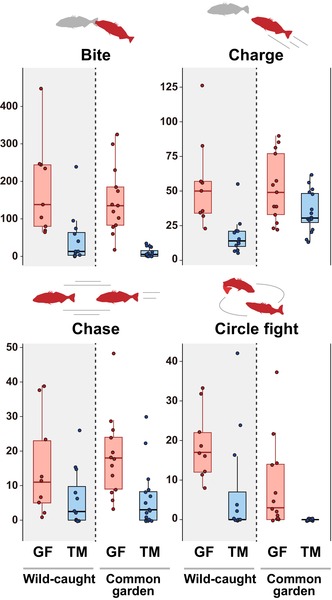

## 1 Introduction

Animals exhibit a great variety of innate behaviors, and aggressive behaviors are used in various ecological and social contexts, including sexual, parental, predator–prey, and territorial conflicts. Although this behavior is widespread throughout the animal kingdom, there is tremendous diversity in aggressiveness even within species and among closely related species (reviewed by King 1973; Lischinsky and Lin 2020; Ostfeld 1990). In particular, the expression patterns of aggressive male behavior in a reproductive territorial context (i.e., reproductive territoriality) can be highly variable, either intra‐or interspecifically, and often have a strong heritable component (e.g., Bakker 1994; Bergeon Burns, Rosvall, and Ketterson 2013; Gaudreault and Fitzgerald 1985; Robertson and Rosenblum 2010; Tynkkynen, Rantala, and Suhonen 2004). Understanding the evolutionary mechanisms of male reproductive territorial behavior is a fascinating topic in evolutionary behavioral ecology (reviewed by Soma et al. 2008). However, compared to many studies on ultimate selective factors, the genetic and molecular bases underlying the evolutionary divergence in reproductive territorial aggressiveness remains largely unexplored in wild animals (Zinzow‐Kramer et al. 2015). Identifying the causal genes and mutations underlying the diversification of complex behavioral traits remains a major challenge, especially in wild animals. However, if we can utilize intraspecific populations/ecotypes or closely related species that show notable phenotypic divergence in the target behavioral traits and no intrinsic postzygotic isolating barriers evolve between them, a forward‐genetic screen including quantitative trait locus (QTL) analysis is a promising approaches and an important first step that elucidates the genetic and molecular mechanisms of the behavioral evolution, together with a comparative brain transcriptomics approach (reviewed by Niepoth and Bendesky 2020; see also Bendesky et al. 2017; Hu et al. 2022).

To address this question, we focused on the threespine stickleback ( *Gasterosteus aculeatus* ), a traditional vertebrate model used in animal behavioral studies (Huntingford and Ruiz‐Gomez 2009). This species has been established as an excellent model species over the past few decades to explore the evolutionary genetic and genomic bases underlying adaptive phenotypic diversification (reviewed by Gibson 2005; Reid, Bell, and Veerama 2021). Although this fish is primarily marine/anadromous, the ancestral marine populations have colonized and adapted to diverse freshwater habitats on multiple continents in the Northern Hemisphere independently (Bell and Foster 1994). Therefore, freshwater populations exhibit extreme diversity in their morphological, physiological, ecological, and behavioral phenotypes (Bell and Foster 1994; Hendry et al. 2013). With these characteristics, QTL mapping has frequently been performed using ancestral marine and freshwater populations for genetic studies on the phenotypic evolution of threespine stickleback (reviewed by Peichel and Marques 2017). However, unlike the frequent applications of this powerful method in morphological evolutionary studies, there are few examples of its application in behavioral evolutionary studies (but see Greenwood et al. 2013; Greenwood and Peichel 2015; Kitano et al. 2009).

The aggressive behavior of the threespine stickleback has long been studied, and this species is known to exhibit three types of aggression: juvenile, dominant, and territorial aggressions (Bakker 1986; van Iersel 1953). In particular, male territorial aggressiveness shows prominent variation among different populations/ecotypes (e.g., Bell 2005; Di‐Poi et al. 2014; Huntingford 1982; Scotti and Foster 2007). Previous studies using brain transcriptome and gene expression approaches have strongly suggested the molecular mechanisms underlying expression in male territorial aggression (e.g., Barbasch et al. 2023; Bukhari et al. 2019; Rittschof et al. 2014; Sanogo et al. 2012). They also revealed an association between aggression level and expression of stress response genes at the population level (Aubin‐Horth, Deschênes, and Cloutier 2012) and divergence in gene expression of specific brain regions among individuals with different levels of aggression (Bell, Bukhari, and Sanogo 2016). However, transcriptional reactions related to aggression‐related genes may contain various types of behavioral plasticity (i.e., such as the detection of intruders, evaluation of intruders, choice of aggressive behaviors, and execution of aggressive behaviors) (Aubin‐Horth and Renn 2009; Bukhari et al. 2017). Therefore, the gene expression profiles obtained from previous studies may represent only a part of a highly dynamic process, and further distinction is needed to differentiate responses to behavior, cognition, and interactions between individuals for the identified candidate genes.

Japanese freshwater threespine stickleback populations have unique characteristics (reviewed by Kitano and Mori 2016; Kokita 2022). Most freshwater populations in North America and northern Europe evolved by the end of the last glacial period (Bell and Foster 1994). Conversely, in Japan, the freshwater colonization of the ancestral marine (anadromous) populations occurred in multiple waves, each of which may reflect different interglacial isolations (Kakioka et al. 2020). Therefore, Japanese stickleback systems differ from those in the Pacific Northwest of North America and Northern Europe in terms of divergence times and histories. Distinct freshwater populations typically have unique phenotypes, including behavioral traits throughout the distribution area; however, partly for the abovementioned reasons and the diversity of ecological habitats, some Japanese populations have intriguing characteristics not found in other populations (Kitano and Mori 2016; Mori 1987). Our pilot observations found two populations (Gifu and Tomakomai; see Materials and Methods) that differed significantly in male territorial aggressiveness among these Japanese freshwater populations and were selected as a model system for this study.

In this study, we performed QTL analysis to explore the genetic architecture of population divergence in male reproductive territorial behavior using the Japanese freshwater threespine stickleback. First, we surveyed the distribution of nests across breeding habitats to reveal the reproductive territorial densities of the two focal Japanese populations in nature. We then assessed the phenotypic and genetic differentiation of male territorial aggression levels between these populations, using both wild‐caught and common‐garden individuals. Finally, QTL mapping using the behavioral phenotypes of an F_2_ hybrid cross between the two populations was performed to identify candidate genetic regions contributing to the divergence in male territorial aggressiveness. By doing so, we were the first to provide a QTL‐based genetic basis for population divergence in male territorial aggressiveness in the threespine stickleback.

## 2 Materials and Methods

### 2.1 Studied Populations

As mentioned above, we used two Japanese freshwater threespine stickleback populations that our simple pilot study predicted would differ significantly in male territorial aggressiveness, for this study (Figure S1). One population, named Gifu (hereafter GF), belongs to one of the two major lineages of the Japanese *G. aculeatus* (see also Ishikawa et al. 2019; Kakioka et al. 2020). This lineage is often called “hariyo” stickleback in Japan (Kitano and Mori 2016; Watanabe, Mori, and Nishida 2003) and is estimated to have colonized freshwater approximately 0.17 Mya and strongly suggested to be the oldest extant freshwater lineage of the species reported to date (Kakioka et al. 2020). Another population, named Tomakomai (hereafter TM), is one of the young freshwater populations in Japan that is not almost genetically differentiated from ancestral marine populations and is phylogeographically nested in marine populations by genome‐wide neutral loci (Kakioka et al. 2020; T. Hosoki, personal communication). These two populations also differ in morphology and body coloration; the latter population is relatively similar to ancestral marine populations, although their body size is smaller than the marine populations (see also Ishikawa et al. 2019).

**FIGURE 1:**
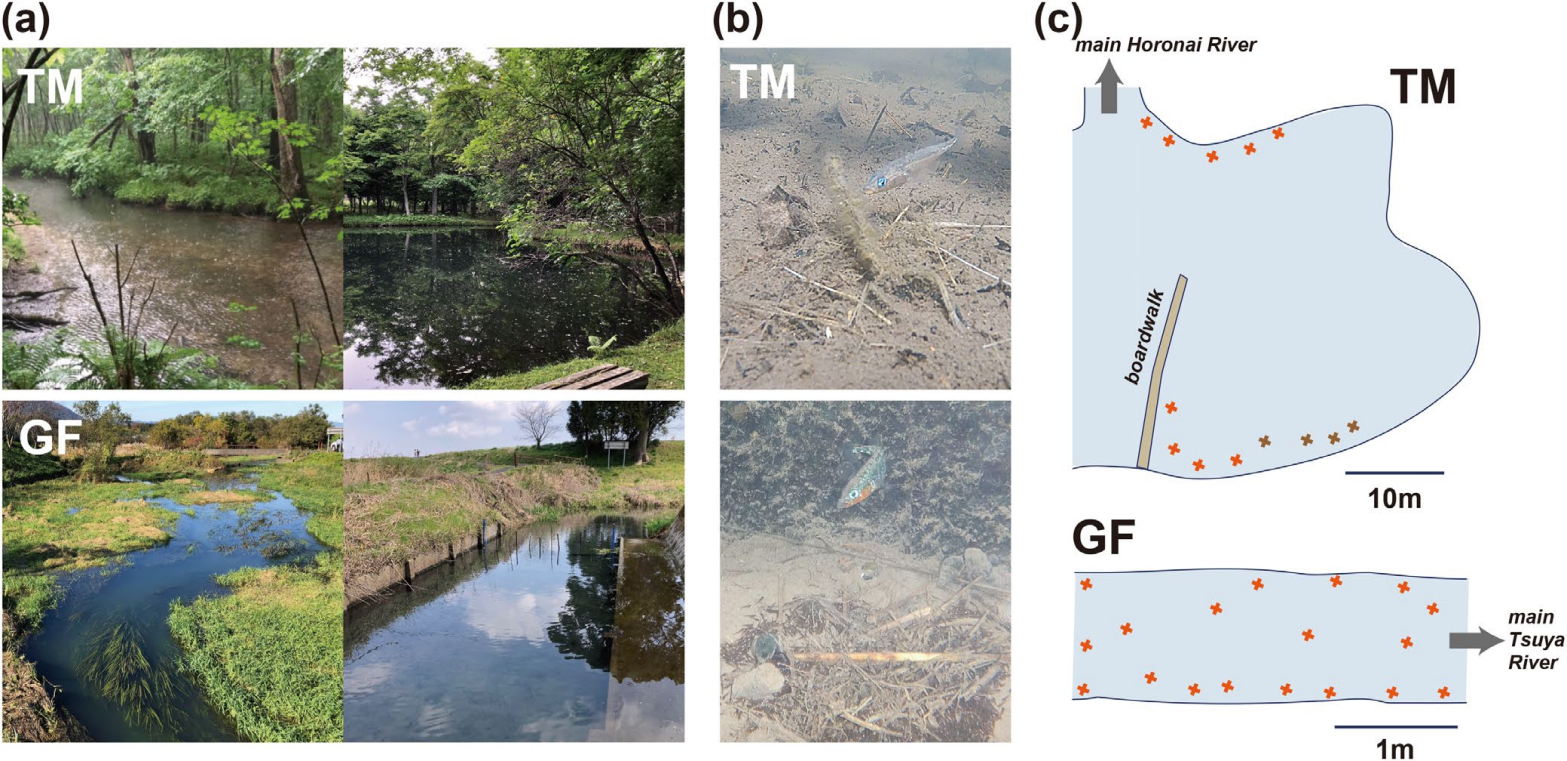
Photographs of (a) riverscapes (left) and breeding habitats (right) in which our field survey was conducted (TM: Tomakomai, GF: Gifu) and (b) breeding of territorial orange‐throated males (see text and Figure S1 for further details). (c) Distribution of male nests across breeding habitats for TM and GF populations.

Although both populations inhabit spring‐fed rivers or streams, the TM population inhabits relatively high‐gradient rivers with faster water velocities than the GF populations (Figure 1a). Therefore, they mainly inhabit shallow areas with low water velocities formed along water bodies, such as ponds connected to the main river. In contrast, the GF populations are found throughout lowland spring‐fed streams in many standing‐water areas. When we roughly calculated the mean river gradient using elevation change and distance data, it was approximately 0.257% for the Horonai River (TM) and 0.037% for the Tsuya River (GF), respectively (see below).

### 2.2 Field Observation of Male Reproductive Territoriality

Observations and recordings of the distribution of male nest sites in the major breeding areas of each population were performed to compare the reproductive territorial densities between the populations in the wild. The survey of the GF population was conducted in a small creek fed by Shimizu Spring Pond and inflowing the Tsuya River, a tributary of the Ibi River system, Gifu Prefecture, Japan (35°15′18″ N, 136°34′01″ E). We also surveyed the TM population in the Horonai River in the Tomakomai experimental forest, Hokkaido University, Hokkaido, Japan (42°40′37″ N, 141°35′38″ E). The survey was conducted on 1 day in the GF population or two consecutive days in the TM population during the main breeding season of each population.

Nesting males and their nests were visually searched for from the shore. Nests were identified and recorded when these males engaged in nesting or parental behaviors, such as sand‐digging, bringing sand, leaves, and branches as nesting material, gluing, and fanning (van Iersel 1953). These observations are straightforward because of the very low turbidity and shallow depths of the breeding areas. When the position of the nests could not be confirmed from the shore, snorkeling observations were also conducted. In addition, we measured the minimum distance from each nest to the nearest nest.

### 2.3 Behavioral Experiments Using Wild‐Caught and Common‐Garden‐Reared Males

For aggressive behavioral assay using the wild‐caught individuals in captivity, the GF mature males with red nuptial coloration were collected using minnow traps from Nakagawa Creek, a different tributary of the Ibi River system from the Tsuya River as mentioned earlier (35°25′34″ N 136°34′41″ E). This population showed nearly year‐round reproduction with two main peaks (spring and autumn) in the breeding season (Mori 1985); therefore, collections were conducted in October–November 2021 and June 2022. Mature TM males were collected from the Horonai River using minnow traps and small stationary nets. The breeding season for this population ranges from March to August (Saito and Nakano 1999); therefore, TM collections were conducted in July 2021 and June 2022. In each population, captured males were kept in 180–240 L tanks under 14:10 h light:dark cycle and at about 18°C with some mature females to maintain reproductive condition. The tanks were filled with 3 ppt salinity‐purified water and independently filtered. Individuals were fed frozen bloodworms (*Chironomus* spp.) daily *ad libitum*. The number of individuals in the acclimation tanks varied depending on the number of captured individuals, with a maximum of approximately 30 individuals, including females. All individuals were acclimated to these tanks for at least 3 days before conducting the behavioral assay.

A one‐on‐one arena experiment for male–male combat was conducted in each population to quantitatively assess population divergence in male territorial aggressiveness. This experiment is essentially based on the “dominance test” described in Bakker and Sevenster (1983) and designed to determine the dominance of two territorial males. In this experiment, we adopted this type of assay to compare the intensity of mutually aggressive interactions between populations. Thus, we had males from the same population compete with each other, and the results were compared between the two populations. This behavioral assay used 60 L (60W × 30D × 36H cm) tanks divided into two compartments by a transparent partition in the central position. Transparent plastic partitions with many small pits allows water molecules to be shared. Each male with red nuptial coloration was placed in a compartment divided by a partition. Males were not provided with sand and nesting materials in this experiment. Before male–male combat, each male was isolated by visual and olfactory contact with another male and maintained for 48 h under the same light and temperature conditions. This timescale is often used by males to establish territories (Tinbergen and van Iersel 1947; van Iersel 1953; see also James and Furukawa 2020). The frozen bloodworms were fed once a day prior to male–male combat. After 48 h, the central partition was carefully removed and the behavior of the two individuals was recorded for 30 min using a video camera (SONY HDR‐CX470) positioned in front of the experimental tank. After video recording, the standard length (SL, mm) and body weight (BW, g) of the individuals were measured. We conducted nine GF and 12 TM experiments, respectively. Individuals used in the arena experiment were not reused. Although two individuals with similar body sizes were selected as closely as possible and used for this assay, some degree of body size difference between the two individuals was inevitable [SL difference: mean and SD (range) = 1.4 ± 1.1 (0.2–3.5) for GF, 1.7 ± 1.5 (0.1–5.8) for TM; BW difference: 0.19 ± 0.09 (0.06–0.32) for GF, 0.14 ± 0.10 (0–0.31) for TM]. However, this degree of difference had little effect on the intensity of aggressive behavior (see RESULTS). The mean SL and BW used in this study were 45.3 (range = 40.7–51.0) and 1.81 (range = 1.25–2.37) for GF population and 49.9 (range = 46.9–56.3) and 1.66 (range = 1.21–2.17) for TM population, respectively. Thus, the GF population had a significantly smaller SL than the TM population (Welch's *t*‐test: *t*_31.6_ = −4.95, *p* = 2.37E‐05; see also Figure S2).

All experimental procedures and housing conditions mentioned above and below followed all applicable national and institutional guidelines, in addition to the care and use of animals.

The first 10 min after the start of the arena experiment was removed for the analysis mentioned below to eliminate any human interference during the setup and allow the individuals to acclimate to the partition removal. The remaining 20 min were analyzed for four territorial aggression‐related traits: “charge” (The male swims very fast toward the opponent), “circle fight/spine fighting” (two males pursue each other rapidly in a circular movement: hereafter “circle fight”), “chase” (attacked animal flees and it releases pursuit in the attacker), and “bite.” These behaviors were defined based on previous research (Tinghitella, Lehto, and Minter 2015; van Iersel 1953). The number of each behavior was counted using JWatcher v1.0 (Blumstein, Daniel, and Evans 2006). It was difficult to distinguish the aggressive behavior expressed by each focal male in the video; therefore, we counted and analyzed the total number of aggressive interactions between them.

Differences in aggressive traits between the two populations were statistically analyzed using a Poisson generalized linear model (GLM). Overdispersion was tested using the *dispersion test* function in the R package AER (Kleiber and Zeileis 2008). The results showed significant (except for “bite” data for wild‐caught individuals) or marginally significant (“bite” data for wild‐caught individuals: *p* = 0.060) overdispersion, and a negative binomial GLM was built and tested the model using the *glm.nb* function in the R package MASS (Venables and Ripley 2002). The greater the body size difference between combating males, the more likely it is to determine their dominance (Rowland 1989); and therefore, body size differences between combating males may influence their aggressive behavior. To account for the potential influence of body size differences between combating males, the difference (|individual_1_ SL‐individual_2_ SL|) was included as a covariate in the model construction. Furthermore, we included the average body size of competing males [(individual_1_ SL + individual_2_ SL) / 2] in the model to assess the effect of absolute body size on the four aggression‐related behavioral components. Indeed, the correlation coefficient between the absolute body size of each individual (i.e., individual_1_ SL or individual_2_ SL) and the average body size was extremely high for both experiments using wild‐caught and common‐garden individuals (see Figure S3). All statistical analyses, including those described below, were performed using R version 4.2.1 (R Core Team 2022).

In addition, a common‐garden experiment was performed to test whether these two populations had heritable divergence in aggressive behavior. We generated four full‐sib experimental crosses for each population using artificial fertilization. Gametes from wild‐caught males and females were used in this procedure. In this study, we followed the standard artificial crossing methods for the threespine stickleback (e.g., Hatfield and Schluter 1996; Leinonen, Cano, and Merilä 2011). In brief, eggs were obtained by gently stripping each ovulated female. Sperm was extracted by chopping the testes with a scalpel from each male, which had been euthanized with an overdose of anesthesia (2‐phenoxyethanol). Artificial fertilization was performed using the eggs and sperm. Fertilized eggs were transferred to Petri dishes filled with purified water at 3 ppt salinity and maintained at 17°C until hatching. Newly hatched larvae were transferred to 30 L tanks (about 50–100 individuals per tank) under 14:10 h light:dark condition at about 18°C. Individuals were fed live *Artemia* nauplii and frozen bloodworms, depending on their size, *ad libitum*. As the individuals from these four crosses grew, they were mixed and kept in the same tank, with their rearing tank size increased to 60–180 L. The aggressive behavioral assay used males over 5 cm SL and with a nuptial color. For each population, 25–30 males were acclimated for at least 1 week in a 180 L tank before the start of the assay. Thus, individuals from the mixed families of each population were randomly selected for the arena experiment. This behavioral assay for common‐garden individuals (GF: *n* = 13; TM: *n* = 16) and subsequent statistical analyses were performed following the same procedures as for wild‐caught individuals. Although two competing males with similar body sizes were selected, there was still some degree of body size difference between them [SL difference: mean and SD (range) = 1.2 ± 1.2 (0.4–4.3) for GF, 1.1 ± 1.6 (0–5.4) for TM; BW difference: 0.18 ± 0.17 (0–0.58) for GF, 0.13 ± 0.14 (0.02–0.45) for TM]. However, this difference had little effect on the intensity of aggressive behavior, similar to findings from experiments conducted on wild individuals (see RESULTS). Their mean SL and BW were 45.3 (range = 42.0–47.7) and 1.64 (range = 1.29–1.98) for GF population and 50.8 (range = 44.5–57.3) and 1.92 (range = 1.19–2.78) for TM population, respectively. Thus, the GF population had a significantly smaller SL than the TM population in the common‐garden experiment (Welch's *t*‐test: *t*_44.3_ = −8.26, *p* = 1.66E‐10; see also Figure S2).

Although we tried to raise the experimental fish under the same rearing conditions as much as possible (water temperature, day length, amount of food, etc.), our experiment does not meet the requirements of a strict common‐garden experiment. During the common‐garden rearing, the stocking density for mixed families differed between the two populations (GF: approximately 0.4 individuals/L; TM: approximately 0.8 individuals/L). Because GF individuals had a slower growth rate, we aimed to accelerate their growth. In addition, the age of individuals used for the arena experiment differed between the two populations (GF population: about 19 months old; TM population: about 13 months old). This age difference is due to the divergence in growth rate; that is, the TM population matured at an earlier age than the GF population. Nonetheless, as mentioned above, the GF individuals used were significantly smaller than the TM individuals (see also Figure S2). Although we acclimated experimental males for at least 1 week before the start of the behavioral assay under a strict common‐garden environment, our common‐garden experiment has some limitations in terms of achieving an ideal design.

### 2.4 Creation of a F_2_ Hybrid Cross and F_2_ Male Behavioral Phenotyping

To perform the QTL analysis of male territorial aggressiveness, we created a full‐sib experimental cross using artificial fertilization. The culture method of fertilized eggs and rearing methods for the progeny were the same as those mentioned above. Gametes from a wild‐caught GF male (F_0_ male) and a wild‐caught TM female (F_0_ female) were used for this procedure. Approximately 50 newly hatched larvae of F_1_ hybrid individuals were transferred to a 30‐L tank. As these individuals grew, the rearing tank was changed to 60 L and raised until they reached approximately 50 mm SL used for the F_2_ hybrid generation. The F_2_ hybrids were created using 10 mature F_1_ hybrid males and 23 egg clutches from multiple F_1_ hybrid females from June to September 2021. As we did not individually identify the females from which the egg masses were collected, the exact number of females and the number of clutches per female used for artificial crossing remain unknown. After hatching, we kept a mean of 50 F_2_ larvae per egg clutch in 10 L tanks. The rearing tanks were then upgraded to 60–350 L tanks after individuals from each family grew and were mixed. They were maintained at a density of 30 individuals in 60 L tanks from May 2022 until the start of the behavioral experiments.

Aggressive behavioral experiments were conducted on 100 F_2_ mature males from September to November 2022. Each experimental male was individually transferred to a 23‐L experimental tank for acclimation when the fish reached > 45 mm SL and exhibited nuptial coloration. Half of the tank's bottom area was covered with 5 cm thick sand (see Figure 3a), and a sufficient amount of boiled palm fibers was provided as nesting material (see Kitano, Mori, and Peichel 2007). Each F_2_ male was kept alone for 2 days, and if nesting behavior was observed, they were kept until nesting was complete. We confirmed nest completion by observing “creeping through” (Sevenster 1961; van Iersel 1953). If nesting behavior was not observed during the 2 days, the individual was considered not to be nesting, and further experiments were discontinued. A behavioral assay was conducted to measure the territorial aggressiveness of each F_2_ hybrid male, following a previous study (e.g., Bakker and Sevenster 1983; Bell 2005; Yong, Lee, and McKinnon 2018), with some modifications. A transparent chamber (15 L × 7D × 7H cm) without holes on the sides was placed on the opposite side of the nesting area (see Figure 3a). We introduced a stimulus of a pure TM or GF male into the chamber and recorded the behavior of a focal F_2_ male on video. The recording lasted for 11 min, with the videographer leaving within 1 min after the recording. To account for the effect of the population of the stimulus male on F_2_ male behavioral responses, a stimulus TM male was presented on the first day after nest completion for each F_2_ male. After video recording, the chamber was removed, and the focal F_2_ male remained in the experimental tank. The next day, a mature stimulus GF male was placed in the chamber, and the behavior of the F_2_ male was recorded on video. After each focal F_2_ male was euthanized with an overdose of anesthesia (2000‐fold diluted 2‐phenoxyethanol), the SL and body weight were measured, and parts of the caudal and pectoral fins were cut and stored in 99.5% ethanol. During the behavioral phenotyping assay for the QTL analysis, we used only one male stimulus for each population to standardize the stimulus. However, another male was used in the TM population because the first male died during the experiment. These stimulus males showed nuptial color, and the body size of TM and GF males were 53.5 or 48.9 mm SL and 49.9 mm SL, respectively.

Behavioral phenotyping of video‐recorded F_2_ males was performed as follows: During the 5 min, once the focal male first oriented toward the stimulus male with binocular fixation, we counted the number of times which the focal F_2_ male bit the surface of the chamber containing the stimulus male (hereafter “number of bites”) and measured the total time (sec) the focal F_2_ male spent within one body length of the stimulus male (hereafter “time near the stimulus”). If the focal males never oriented toward the stimulus male within 6 min after introducing the stimulus male, the males were excluded from further analyses. In addition, the time spent near the stimulus was set to zero if the focal males oriented toward the stimulus.

### 2.5 QTL Mapping for Male Territorial Aggressiveness

The genomic DNA of two F_0_ individuals (a GF grandfather and a TM grandmother) and their 100 F_2_ progeny were extracted from the pectoral or caudal fins using a DNeasy Blood & Tissue Kit (Qiagen). Double‐digest restriction site‐associated DNA sequencing (ddRAD‐Seq) libraries for these 102 individuals were prepared using EcoRI and BglII (New England Biolabs), according to Peterson's protocol (Peterson et al. 2012), with slight modifications (Sakaguchi et al. 2015). These libraries were sequenced on the Illumina HiSeq × Ten with the 150‐bp paired‐end protocol at Macrogen, Japan. The RAD‐Seq reads (755,819,850 reads, 114 Gb of data; see Table S1) were filtered using fastp v0.22.0, to remove the Illumina adapter and eliminate low‐quality regions. The trimmed reads were mapped to the reference genome of the threespine stickleback genome assembly (v5) (Peichel et al. 2020) using bowtie2 v2.5.1. Single‐nucleotide polymorphism (SNP) calling was performed using the *ref_map.pl* pipeline of Stacks v2.64 (Rochette, Rivera‐Colón, and Catchen 2019), and genotyping of the F_2_ population was performed using the *populations* program of Stacks. The SNP calling identified 4404 markers, which were subsequently filtered and used for linkage map construction (see below).

Linkage map construction of this hybrid cross was performed using JoinMap v4.0 (van Ooijen 2006), as described by Kakioka et al. (2013). In this study, we used F_2_‐type RAD‐SNP markers, which were genotyped more than 85% of the progeny and did not show significant segregation distortion (χ^2^ test, *p* < 0.001). Linkage groups (LG) were identified with an independence logarithm of odds (LOD) threshold of 6. Unlinked markers and small LG, including fewer than three markers, were excluded from further analyses. A linkage map was built using a regression mapping algorithm, with a recombination frequency of less than 0.4. Up to three rounds of marker positioning were conducted, with a jump threshold of 5. Rippling was performed after the addition of each new marker. The map distances were calculated using the Kosambi's mapping function. The initial mapping identified potential errors that appeared as doubtful double recombinants using the genotype probability function of JoinMap (*p* < 0.001). Therefore, these suspicious genotypes were considered missing values. A linkage map was then constructed using the corrected dataset. Potential error elimination and linkage map construction were iterated until no dubious genotype was identified, removing markers with > 20% missing value or distorted (χ^2^ test, *p* < 0.001) in each iteration. The R package LinkageMapView (Ouellette et al. 2018) was used to draw the linkage map.

The QTL analysis was performed using the R/qtl package (Arends et al. 2010; Broman et al. 2003). In this analysis, *G. aculeatus* sex chromosome LG19 was treated as an X chromosome. Four behavioral datasets, that is, the number of bites on a stimulus TM or GF male and the time spent near the stimulus, were adopted as phenotypic traits for this study. First, genotype probabilities were estimated using the *calc.genoprob* function. For binary and quantitative traits, interval mapping was performed using the EM algorithm with a binary model using the *scanone* function. We included the body size difference between the focal F_2_ males and a stimulus TM or GF male, as well as the date on which the behavioral assay was carried out, as additive covariates in the QTL analysis. The latter data were set as the number of days that elapsed since the first experimental start date (i.e., the first experiment date was zero). Because we used two stimulus males in the TM during the experiment, as mentioned above, we also added individual ID (i.e., TM1 or TM2) when a TM male was presented in the chamber as an additive covariate. The significant thresholds of LOD scores were determined by 1000 genome‐wide permutations (genome‐wide significance threshold: α = 0.05). The 90% and 95% Bayesian credible intervals (CI) of significant QTL were calculated using the *bayesint* function. The percentage of phenotypic variance explained (PVE) by significant QTL was calculated using the *fitqtl* function.

## 3 Results

### 3.1 Reproductive Territorial Pattern in the Wild

During field observations, we found 13 nesting territories defended by males across the TM breeding habitat (2‐day observation) and 27 nesting territories across the GF breeding habitat (single‐day observation) (Figure 1b,c). In the TM habitat, males bred in the nearshore part of the waterbody, similar to a pond connected to the main river, although the offshore habitat of this waterbody maintained a shallow and flatter bottom. Conversely, male nesting territories existed across the GF breeding habitat, resulting in divergent nest distribution patterns. Additionally, there was a large difference between the populations in the size of the observation areas (5 × 1.5 m in the GF population and 35 × 35 m in the TM population). Thus, the density of reproductive territories was much greater in GF (3.6 nests/m^2^) than in TM (0.01 nests/m^2^). In addition, the distance between all nests was within 1 m in the GF population, whereas it ranged from 1.5 m to a maximum of 5.6 m in the TM population. These results indicated that the distribution of reproductive territories differed greatly across breeding habitats between the two focal Japanese populations.

### 3.2 Genetic Population Divergence in the Intensity of Male Territorial Aggression

**FIGURE 2:**
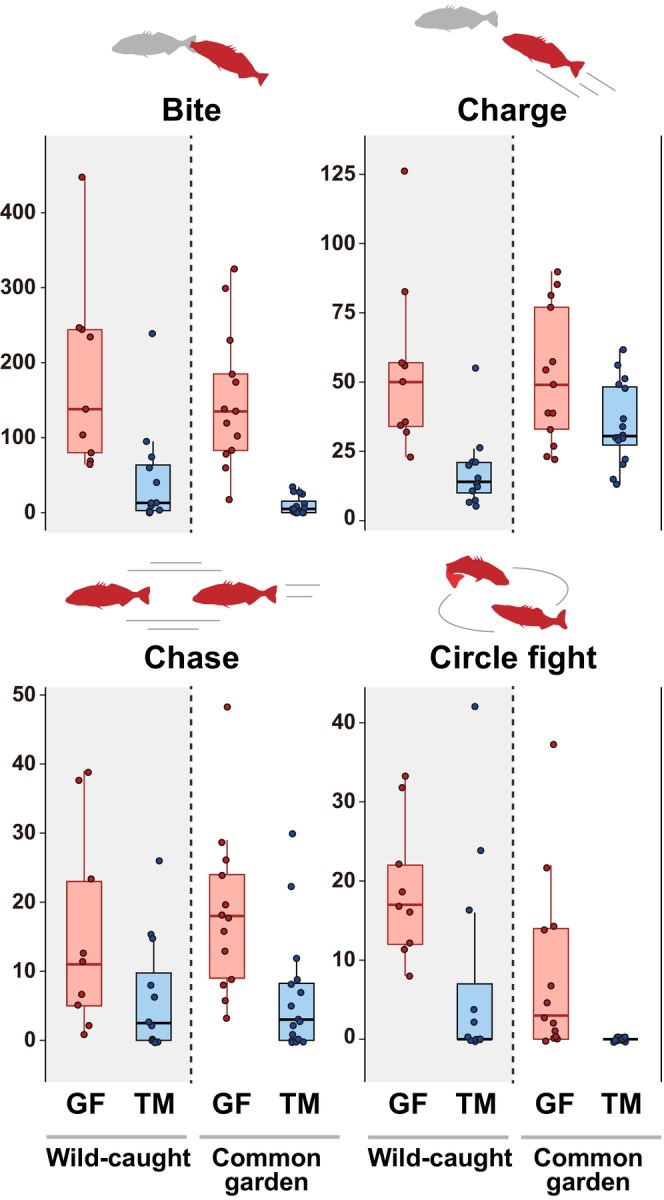
Expression levels of four aggression‐related behavioral components (the numbers of bites, charges, chases, and circle fights) in the one‐on‐one arena experiment using each GF and TM population. Results using both wild‐caught and common‐garden males were shown. Each box's lower and upper limits correspond to the first and third quartiles, and the horizontal line shows the median. Whiskers extend to the lowest and highest observed biases within 1.5 interquartile range units from the box.

Our behavioral experiments using wild‐caught males revealed that GF males exhibited significantly higher levels of aggression, particularly in terms of bite, charge, and circle fight than TM males (Table 1; Figure 2). While there were no statistically significant differences between these populations for chase, the higher trends in aggressive behavior of the GF males were observed. A similar pattern was observed for males reared in the common garden (Table 1; Figure 2). The GF males demonstrated significantly higher levels in bite, charge, and chase. Circle fight was observed in many GF males, but not in TM males. Thus, the differences in the aggression levels between these populations remained consistent in the common‐garden experiment.

**TABLE 1:** Parameter estimates from the negative binomial GLM conducted to examine population differences (TM vs. GF populations) in four aggression‐related behavioral components (the numbers of bites, charges, chases, and circle fights) in the one‐on‐one arena experiment.

	Wild‐caught individuals				Common‐garden individuals			
	Estimate	Standard error	*Z*	*p*	Estimate	Standard error	*Z*	*p*
**Bite**								
Intercept	2.969	5.196	0.571	0.568	0.147	3.695	0.040	0.968
Population	−1.806	0.722	−2.503	0.012	−3.418	0.612	−5.582	2.38E‐08
|SL difference|	0.047	0.247	0.191	0.849	−0.018	0.149	−0.118	0.906
Average SL	0.050	0.118	0.421	0.673	0.108	0.082	1.324	0.186
**Charge**								
Intercept	2.703	2.493	1.084	0.278	2.094	1.448	1.446	0.148
Population	−1.307	0.347	−3.762	0.0002	−0.645	0.240	−2.684	0.007
|SL difference|	0.003	0.120	0.028	0.978	−0.004	0.059	−0.072	0.943
Average SL	0.029	0.057	0.514	0.608	0.041	0.032	1.289	0.198
**Chase**								
Intercept	3.476	5.839	0.595	0.552	1.870	3.570	0.524	0.600
Population	−0.778	0.811	−0.958	0.338	−1.244	0.590	−2.109	0.035
|SL difference|	−0.015	0.280	−0.055	0.956	−0.090	0.148	−0.610	0.542
Average SL	−0.016	0.133	−0.124	0.902	0.026	0.079	0.324	0.746
**Circle fight**								
Intercept	−12.864	5.657	−2.274	0.023	11.920	3.697	3.225	0.001
Population	−3.643	0.799	−4.560	5.12E‐06	−19.218	1284.397	−0.015	0.988
|SL difference|	0.006	0.267	0.022	0.983	−0.033	0.086	−0.383	0.701
Average SL	0.356	0.129	2.768	0.006	−0.217	0.082	−2.650	0.008

*Note:* Population effect here refers to the TM population. |SL difference| indicates the absolute value in body size (SL) differences between two combating males (i.e., |individual_1_ SL‐individual_2_ SL|). The average SL reveals the average body size (SL) of two combating males [i.e., (individual_1_ SL + individual_2_ SL) / 2].

No significant correlation was observed between the difference in body size between combating males and the intensity of aggressive behavior in any of the experiments (Figure S4; see also Table 1). In addition, there was no significant effect of the average body size of competing males on the overall level of aggression, except for a few aggression‐related behavioral components (Table 1; Figure S5).

Thus, these results showed that male territorial aggressiveness differed genetically between populations.

### 3.3 Behavioral Phenotypic Variance in F_2_ Hybrid Males

**FIGURE 3:**
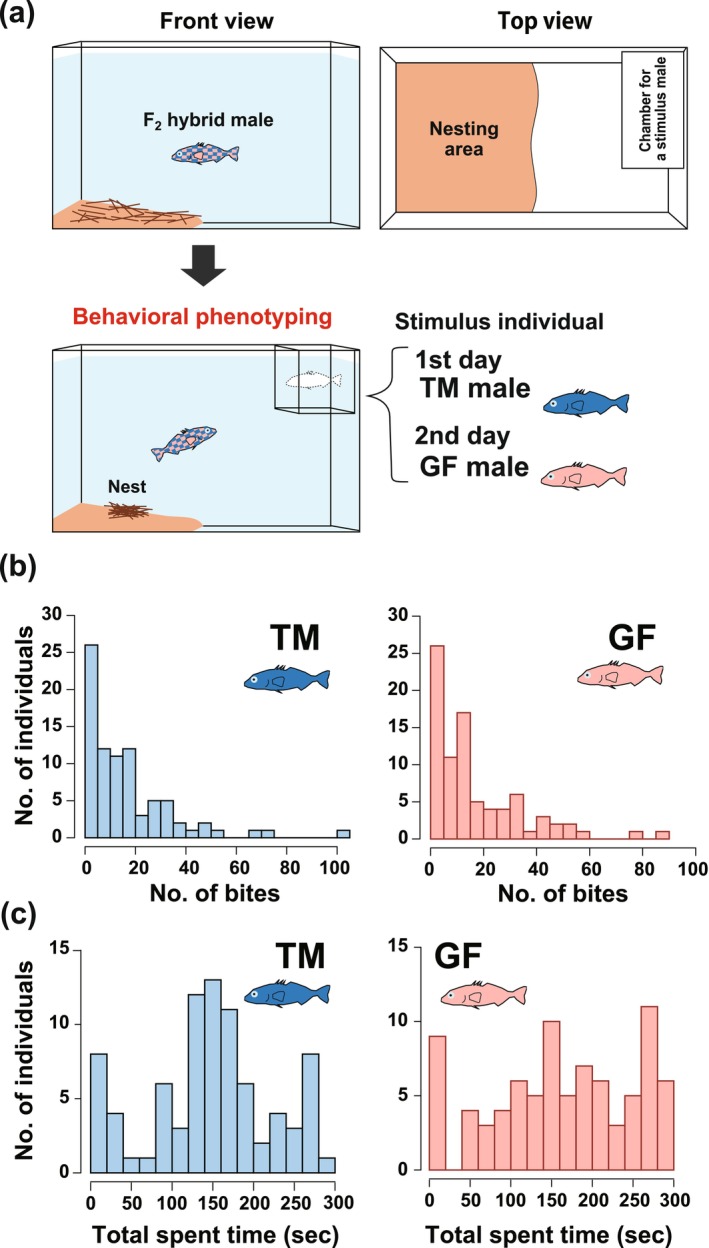
(a) Schematic illustration of behavioral phenotyping for the QTL analysis. Phenotypic distribution of two aggression‐related behavioral components, that is, (b) the number of bites toward each TM or GF stimulus, and (c) the total time (sec) spent near the stimulus, in F_2_ progeny derived from the GF and TM founder individuals.

Among the 100 mature males of the F_2_ hybrid used in the behavioral experiments, some focal males did not orient to each stimulus male within 6 min of the start of the experiment. We excluded these males from the analysis of the TM‐stimulus and GF‐stimulus experiments. As a result, we obtained datasets from 83 (TM‐stimulus) and 84 (GF‐stimulus) males for further analyses, respectively (Figure 3b,c). When the stimulus TM male was presented, the number of bites and time near the stimulus ranged from 0 to 103 times (mean and SD = 16.4 ± 18.1) and 0 to 290 s (147.0 ± 77.1), respectively. In the case of GF‐stimulus, they ranged from 0 to 89 times (17.2 ± 17.8) and 0 to 297 s (164.3 ± 87.3). Thus, the phenotypic values of the F_2_ individuals varied considerably.

For 75 focal males oriented to both stimuli males, the mean (± SD) number of bites and time near the stimulus was 15.1 ± 15.4 and 145.0 ± 74.3 toward the TM‐stimulus, and 18.2 ± 18.2 and 162.9 ± 87.5 toward the GF‐stimulus, respectively. The time spent near the GF‐stimulus of the focal males was significantly longer than that near the TM‐stimulus (paired *t*‐test, *t*_74_ = −2.2311, *p* = 0.029), and a similar trend (but not significantly) was detected for the number of bites (*t*_74_ = −1.5837, *p* = 0.118). A significant correlation between the aggressive intensity of the focal males toward the TM‐stimulus and GF‐stimulus was detected for analysis using these 75 focal males (Pearson's *r* = 0.51, *p* = 3.8E‐6 for number of bites; *r* = 0.65, *p* = 2.9E‐10 for time near the stimulus; Figure S6). Thus, the aggressive responses of focal F_2_ males toward the stimulus males were consistent regardless of the stimulus populations.

### 3.4 A QTL Underlying Divergence in Male Territorial Aggressiveness

A final linkage map was constructed using 980 ddRAD SNP markers from 100 F_2_ hybrid males. This map consisted of 21 LG corresponding to the haploid chromosome number of the threespine stickleback (Figure S7). The total length of the linkage map was 1330 centimorgan (cM), with a mean marker distance of 1.36 cM.

**FIGURE 4:**
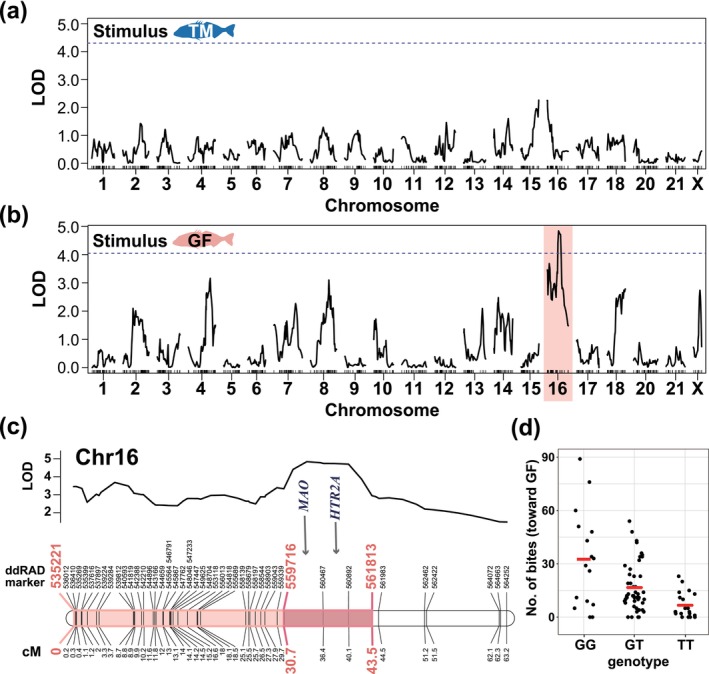
LOD score curve for the QTL analysis of the F_2_ male aggression level (the number of bites) toward the stimulus (a) TM or (b) GF male. A dotted horizontal line shows the significance level (95%). Sex chromosome 19 was treated as an X chromosome (see text). (c) Detailed LOD score plot across chromosome 16 and the locus position for the male aggression level toward the stimulus GF male detected in the analysis. The 95% (light color) and 90% (dark color) Bayesian confidence intervals for the locus are shown, respectively. The ddRAD‐marker positions are shown with centimorgan (cM) data. Arrows indicate the positions of two candidate genes (*MAO* and *HTR2A*; see also text). (d) Effect plot of genotype on the number of bites toward the stimulus GF male at the chromosome 16 QTL. The raw data are plotted with the mean value. The genotypes are GG for homozygous GF (*n* = 16), TT for homozygous TM (*n* = 20), and GT for heterozygous GF/TM alleles (*n* = 48), respectively.

QTL analysis was conducted using the two behavioral phenotypes and the abovementioned linkage map (Figures 4a,b and S8). There were no significant peaks in the time near each stimulus male (Figure S8). However, a single significant peak of the LOD score, containing two SNP markers (560467 and 560892), was detected on LG16, that is, chromosome 16 (Chr16) in the number of bites toward only the stimulus GF male (highest LOD peak = 4.84, *p* = 0.017; Figure 4b,c). This locus explained 20.8% of the observed phenotypic variance. Effect plots of the QTL revealed that alleles from the GF population increased the number of bites (Figure 4d).

The 95% Bayesian CI (from 0 cM to 43.5 cM of Chr16) and 90% CI (30.7–43.5 cM) for the highest LOD peak contained the ensemble‐annotated 611 genes and 63 genes in the threespine stickleback genome assembly (v5), respectively (Table S2). Two notable behavior‐related genes, *MAO* (monoamine oxidase; ENSGACG00000009353) and *HTR2A* (5‐hydroxytryptamine receptor 2A; ENSGACG00000008088), which are involved in the serotonin and dopamine synthesis pathways, were present not only at the 95% CI but also at the 90% CI (Figure 4c).

## 4 Discussion

Aggressive territorial behavior in male threespine stickleback is a traditional research topic that has been studied for many years (Bakker 1986; Huntingford and Ruiz‐Gomez 2009; ter Pelkwijk and Tinbergen 1937). In this study, we conducted ecological genetic analyses, including the first QTL mapping of territorial aggression levels in threespine stickleback males, using two Japanese freshwater populations predicted to differ in territorial aggression. Our behavioral experiments using wild‐caught and common‐garden individuals revealed a signal of genetic divergence in male territorial aggressiveness between the GF and TM populations. GF males exhibited higher levels of territorial aggression than TM individuals in all behavioral components measured, although some components did not show statistically significant differences. In general, larger threespine stickleback males are more aggressive and have an advantage in dominance contests (Mehlis et al. 2010; Rowland 1989); however, this is not the case in some populations (i.e., no correlation between male body size and aggressiveness; Bakker 1986; van den Assem 1967). Indeed, GF males with higher levels of aggression were smaller than TM males. Additionally, our GLM analysis, by incorporating individual body size data, supported the aforementioned results in both wild‐caught and common‐garden individuals. Thus, the difference in aggressiveness between GF and TM is subject to strong genetic determinants although we cannot completely rule out the unforeseen effects of body size differences between populations. In particular, there was extreme divergence in the number of bites under common‐garden rearing. This phenomenon may be one reason why significant loci were found in the number of bites in the QTL analysis (see below).

However, there are some caveats to our experiments. As mentioned earlier, our common‐garden experiment has some limitations in terms of achieving an ideal design. First, during the common‐garden rearing, the stocking density differed between the two populations in order to promote the growth of GF individuals, which had a slower growth rate. Second, the age in months of individuals used for the arena experiment differed between the two populations. Although Lachance and Fitzgerald (1992) found no relationship between age and aggression level in male threespine stickleback, this potential age effect may not be completely negligible. Third, the relatedness structure of the common‐garden individuals used in the arena experiment was unknown because we mixed multiple full‐sib crosses from each population and housed them in the same tank. This was primarily due to spatial constraints in the fish‐rearing room and limitations on tank size and number. Additionally, in the arena experiment, we conducted only one trial per male, meaning we did not assess the repeatability of phenotypic values at the individual level. As Bolyard and Rowland (2000) suggested that past experiences of winning or losing can affect subsequent aggression levels in threespine stickleback, we limited the experiment to one trial per individual to avoid potential confounding effects. Therefore, we must note that the individual's behavioral phenotype we measured may include indirect genetic effects, specifically the influence of the partner male's behavior in each trial. The indirect genetic effect is generally thought to act additively or synergistically in coordination with the direct genetic effect (Lane, Wilson, and Briffa 2020), and the influence of indirect genetic effects on behavioral variation has been reported in many studies (reviewed by Bailey, Marie‐Orleach, and Moore 2018). It has also been suggested that aggression can change through interactions with other individuals (Moore, Brodie, and Wolf 1997; Wilson et al. 2009). Thus, we concluded, with some caveats, that the GF and TM populations genetically diverge in male territorial aggressiveness.

What were the ultimate factors leading to the evolution of different territorial aggressiveness? This study did not aim to explore these factors. However, we speculated why these populations might differ in this fitness‐related behavioral trait and considered several hypotheses. First, our field observations of the distribution of male nest sites in the major breeding areas of each population revealed that the territorial patterns (i.e., territorial density and distance between nesting territories) significantly differed between the populations, although our survey area and duration were limited. The territorial colonies of the GF population were dense and many males did not maintain their territories (see also Mori 1993). This suggests that GF males are exposed to strong competition for reproductive territorial acquisition because of their smaller breeding areas. High population densities generally lead to increased reproductive territoriality for resource acquisition and defense (Barrero et al. 2024; Schoener and Schoener 1980; Yoon et al. 2012). This pattern occurs because defending the territory is more important than attracting females, which increases reproductive success in competitive environments (Warner and Hoffman 1980). Previous studies have suggested that males with greater fighting abilities are more likely to breed successfully in high‐density environments (e.g., Bertin and Cézilly 2005; Zeh 1987). Thus, the higher aggressiveness of the GF population is consistent with the results of previous studies, emphasizing its effect on population density.

Second, differences in behavior may be influenced by different predation pressures between these populations. While no predatory aquatic animals were found in the GF habitat, many brown trout ( *Salmo trutta* ) individuals, which are invasive and generalist piscivores, inhabited the TM habitat. Although we do not have accurate information on the invasion period of brown trout into the TM habitat, this species often outcompetes and eats native fishes (Sánchez‐Hernández 2020). Indeed, our stomach content analysis of large brown trout individuals confirmed their predation on adult threespine stickleback (Figure S9). Aggressive male–male interactions may increase the risk of predation on males or damage to and disturbance of nest sites by attracting the attention of predators (Scotti and Foster 2007). In comparative studies using threespine stickleback ecotypes under different predation pressures, ecotypes with lower predation pressures tended to be more aggressive (e.g., Giles and Huntingford 1985; Huntingford 1982; Lacasse and Aubin‐Horth 2012; Tulley and Huntingford 1988). Thus, strong predation pressure from invasive piscivorous fish species may have lowered the territorial aggressiveness of the TM population through rapid adaptive evolution.

Third, although the abovementioned hypotheses focused on ecological factors contributing to the genetic differentiation of territorial aggressiveness between populations, a neutral evolutionary process involving a combination of *de novo* mutations related to behavioral phenotypes (or standing genetic variation harbored by ancestral marine populations) and random genetic drift may occur rather than natural selection. In particular, the GF population is the oldest freshwater‐colonized population of this species reported to date, as mentioned above (Kakioka et al. 2020); therefore, this possibility is not be negligible. Further detailed investigations are needed to determine the ultimate factors underlying territorial aggressive territorial divergence, including other factors.

There are still many unknown aspects of the genetic architecture of behavioral variation, including aggressive territorial behavior, in wild animals. However, in stickleback fishes, some behavioral QTL, such as courtship behavior (Kitano et al. 2009), schooling behavior (Greenwood et al. 2013; Greenwood and Peichel 2015), and boldness (Laine et al. 2014) have been previously identified. In this study, we detected a significant QTL locus underlying evolution at the aggressive territorial level in stickleback fish. The F_2_ male behavioral phenotyping for QTL analysis was designed differently from the arena experiment. This difference arises because the arena experiment measured the total number of aggressive behaviors between two males, which is unsuitable for evaluating the phenotype of each F_2_ males. However, both behavioral assays have been widely used in previous studies to quantify territorial aggression, particularly in terms of bite frequency (e.g., Bakker and Sevenster 1983; Bell 2005; Yong, Lee, and McKinnon 2018; James and Furukawa 2020; Rowland 1989). Therefore, both methods are thought to essentially assess similar characteristics related to the level of territorial aggression. The significant QTL identified was related to the difference in the number of bites between the populations. However, this was detected only when a GF male was used as the stimulus. There are two possible reasons why significant loci were not detected for the number of bites when the TM male stimulus was presented. First, the order of stimulus presentation might have influenced the results because we always presented TM individuals to focal F_2_ individuals on the first day and GF individuals on the second day. Another possibility is that the population‐specific phenotypic differences in the presented stimulus. Although we did not quantitatively measure these phenotypes, the stimulus GF male always expressed stronger nuptial coloration and actively moved in the chamber. These characteristics may have led the experimental F_2_ males to perceive the GF‐stimulus as a more threatening intruder and become more aggressive. In addition, the overall bite level of the experimental F_2_ males was not significantly different between the TM and GF‐stimulus, whereas the F_2_ males with higher bite values tended to bite more when the stimulus male was the GF. This phenomenon could be why a significant QTL was detected only in the case of the stimulus GF males. On the other hand, our correlation analysis of the number of bites by F_2_ males in response to the GF and TM stimulus individuals revealed a significant positive correlation between the two stimuli. Therefore, it is considered that the F_2_ male behavioral phenotypes reflect, to some extent, the population differences in aggression levels regardless of the stimulus population.

In this study, no QTL was detected for the time F_2_ males spent near each stimulus male. Although the time spent near the stimulus showed a significant positive correlation between the GF and TM stimuli, this trait may not necessarily reflect differences in the level of aggression between the two populations. For example, the time spent near the stimulus may increase even if only less aggressive males approached and watched the intruder.

Variations in behavior are generally known to be largely polygenic (reviewed by Bendesky and Bargmann 2011). Indeed, each of the detected behavioral QTL had a small effect (PVE: 3%–7%) in a QTL study using ninespine stickleback ( *Pungitius pungitius* ) (Laine et al. 2014). Although not an example of wild animals, Zhang et al. (2022) identified several peaks and candidate genes associated with the inter‐strain differences in aggressiveness in the Siamese fighting fish ( *Betta splendens* ) by genome‐wide association study. In this study, we detected a QTL explaining 20.8% of the PVE, which could have a moderate effect on aggressive divergence. Of course, a well‐known phenomenon, the Beavis effect (Beavis 1994), which overestimates QTL effect sizes, cannot be ignored, especially when the sample size is not sufficiently large, as in this study. It has been reported that the power of QTL detection remains almost the same when the marker distance is less than 10 cM (Darvasi et al. 1993). The mean marker distance of our constructed linkage map was 1.36 cM, suggesting that our linkage map is sufficient for QTL detection from the perspective of marker density. Therefore, this locus is considered a suitable genomic region for screening the causal gene (genes) related to aggressive behavior.

A significant QTL locus was found on chromosome 16. We found two notable behavior‐related genes near this locus, *MAO (MAO‐A)* and *HTR2A*, which were included in the 63 genes within the 90% CI. *HTR2A* encodes a serotonin receptor (serotonin 2A receptor), and *MAO* is involved in the metabolism of biogenic amines, including the key neurotransmitters serotonin, noradrenaline, and dopamine (reviewed by Shih and Thompson 1999). Previous studies have suggested that dopamine and serotonin are associated with aggressive behavior (reviewed by Dragovich and Borinskaya 2019; Miczek et al. 2001; Nelson and Chiavegatto 2001). The sex steroid hormone (androgen) is often associated with aggressive male behavior as a reproductive strategy (reviewed by Giammanco et al. 2005; Rubinow and Schmidt 1996), and 11‐ketotestosterone, which is the most important androgen in most fishes, increases significantly in threespine stickleback males when they establish territories and nests (Páll, Mayer, and Borg 2002). However, this genomic region has no well‐known androgen synthesis or androgen signaling pathway genes.

Therefore, *MAO‐A* and *HTR2A* have been proposed as candidate genes that strongly influence aggressive behaviors (Popova 2008). Studies in mammals (humans and mice) have reported that changes in serotonin levels caused by variations in MAO‐A levels in the brain are associated with social behaviors such as anxiety, other emotions, and aggression (reviewed by Kolla and Bortolato 2020; Shih, Chen, and Ridd 1999). Additionally, in Mexican Tetra ( *Astyanax mexicanus* ), the same mutated allele of the *MAO* gene has been found in several cave ecotypes, some of which have evolved independently (Elipot et al. 2014). The allele reduces MAO activity and increases serotonin levels in the cave ecotypes compared to the ancestral surface ecotypes; thereby, it may be partly the molecular basis for the loss of aggression in cave ecotypes (Elipot et al. 2014). Furthermore, a recent study reported that the transfection and overexpression of *MAO‐A* gene in the diencephalon of mature male three‐spined stickleback increased territorial aggression associated with a decrease in serotonin, which is enzymatically cleaved by the MAO‐A enzyme (James and Bell 2021). Thus, the *MAO‐A* gene is an important candidate gene influencing aggressive behavior in the three‐spined stickleback. For another noteworthy gene, *HTR2A*, its polymorphism was reported to modulate aggression in humans (Banlaki et al. 2015; Erjavec et al. 2022), although it remains unclear whether this is a general pattern. In addition, a freshwater threespine stickleback population showed higher levels of territorial aggression and *HTR2A* gene expression than the marine population (Di Poi et al. 2016). Therefore, these two genes are good candidates for identifying the causal gene (genes) underlying the divergence in male territorial aggressiveness between the analyzed populations. Further experimental analyses, including brain region‐specific gene expression and functional mutations in the coding region, are required.

This study revealed a signal of genetic divergence in aggressive territorial behavior between two Japanese freshwater populations of threespine stickleback and gained insight into the genetic basis underlying the population divergence. This highlights the importance of expanding the scope of research to include other populations, which may help us to understand the complex ecological and genetic patterns and mechanisms of adaptive diversification of animal aggressive behavior in the wild.

## Author Contributions

**Haruka Yamazaki:** data curation (lead), formal analysis (lead), funding acquisition (supporting), investigation (lead), methodology (lead), resources (equal), visualization (lead), writing – original draft (equal), writing – review and editing (supporting). **Seiichi Mori:** conceptualization (supporting), investigation (supporting), methodology (supporting), resources (equal), visualization (supporting). **Osamu Kishida:** investigation (supporting), resources (equal). **Atsushi J. Nagano:** investigation (supporting), resources (supporting). **Tomoyuki Kokita:** conceptualization (lead), data curation (supporting), funding acquisition (lead), investigation (supporting), methodology (supporting), project administration (lead), supervision (lead), writing – original draft (equal), writing – review and editing (lead).

## Conflicts of Interest

The authors declare no conflicts of interest.

## Supporting information


Appendix S1.

Appendix S2.

## Data Availability

Raw data of the behavioral experiments and phenotyping/genotyping for the QTL analysis are available as supporting information (Tables S3–S5). The RAD‐seq sequence data are available in DDBJ Sequence Read Archive (DRA) (accession no.: DRR595078–DRR595179).
