# Supplementary material for: QTL‐Based Evidence of Population Genetic Divergence in Male Territorial Aggressiveness of the Japanese Freshwater Threespine Stickleback
Source: Ecol Evol. 2025 Jan 9;15(1):e70795. doi: 10.1002/ece3.70795 (PMC11717901; doi:10.1002/ece3.70795)
Supplement: Supplementary file 1 — Appendix S1. [file ECE3-15-e70795-s002.pdf]

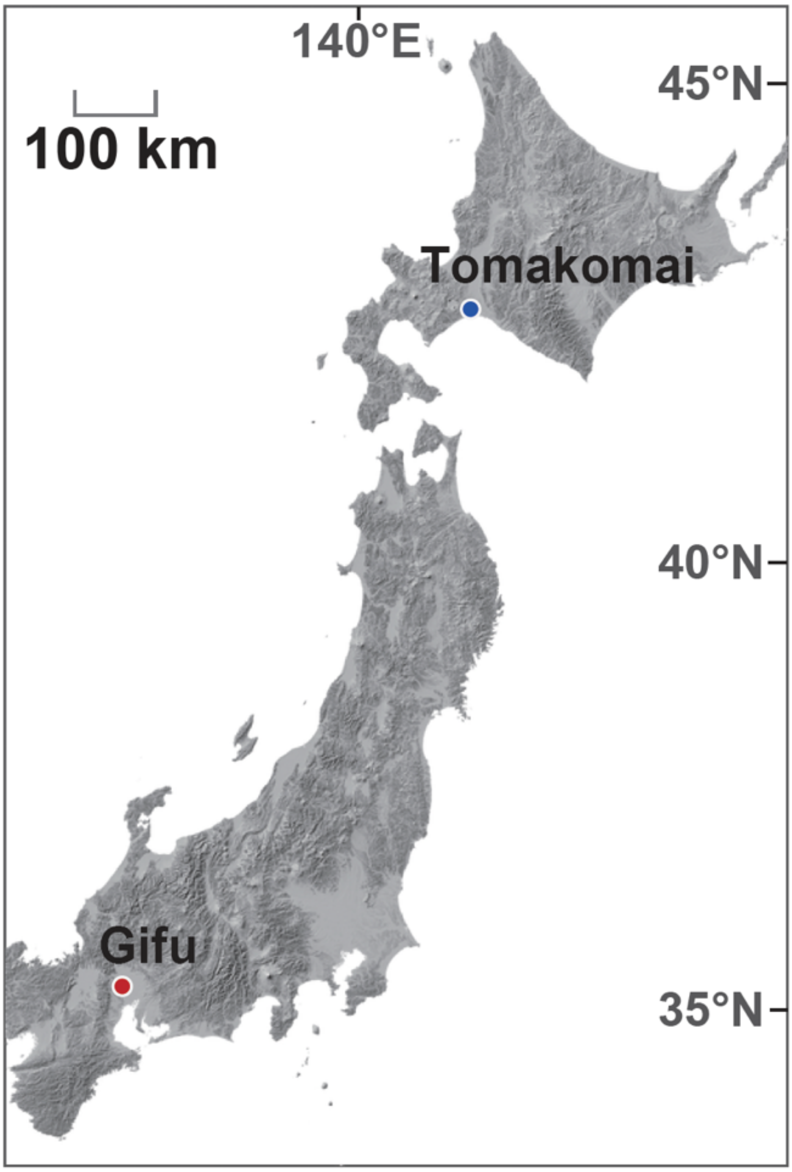

FIGURE S1 Map showing two populations used for this study.

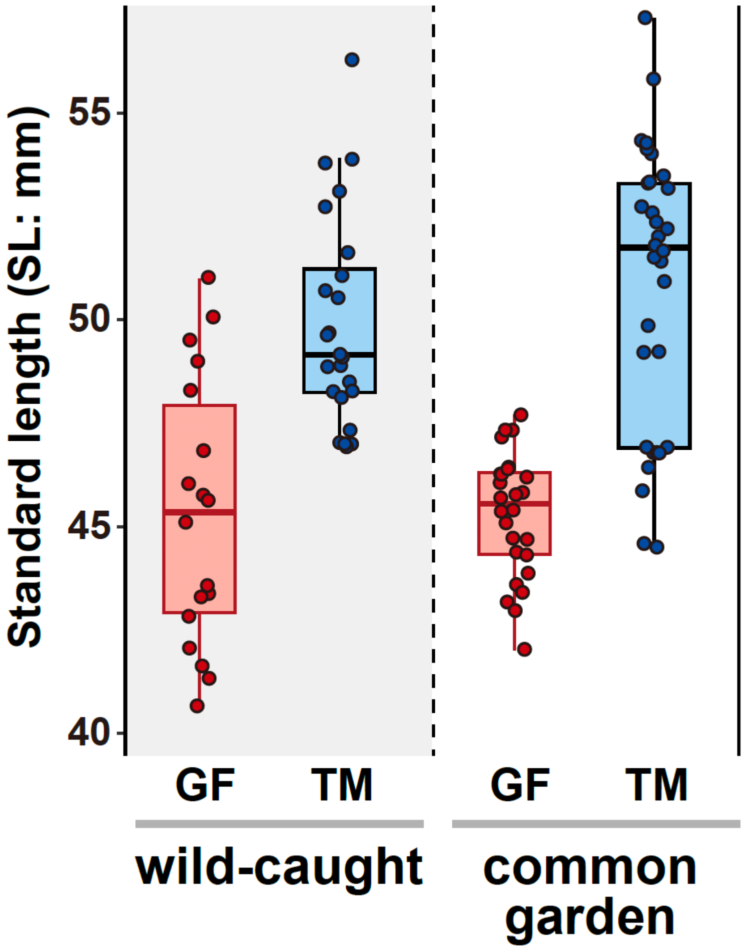

FIGURE S2 Body size (standard length) of GF and TM males used in the one-on-one arena experiment. Data of both wild-caught and common-garden males were shown. Each box's lower and upper limits correspond to the first and third quartiles, and the horizontal line shows the median. Whiskers extend to the lowest and highest observed biases within 1.5 interquartile range units from the box.

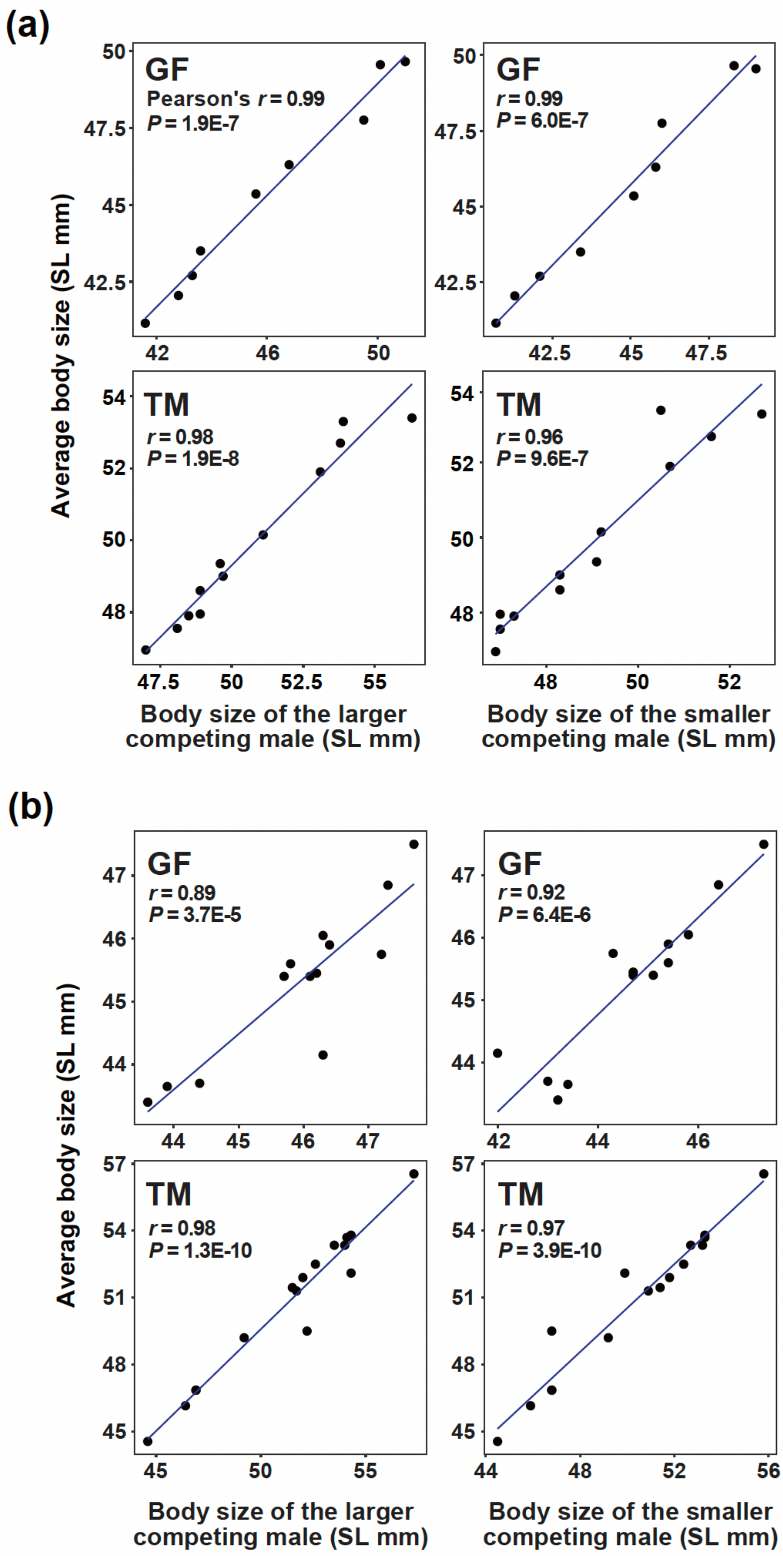

FIGURE S3 The relationship between the absolute body size of each competing male (i.e., larger competing male or smaller competing male) and the average body size of the two males in the one-on-one arena experiment using each GF and TM population. Results using (a) wild-caught and (b) common-garden males were exhibited, respectively. In each analysis, Pearson's correlation coefficient was shown with the *P*-value.

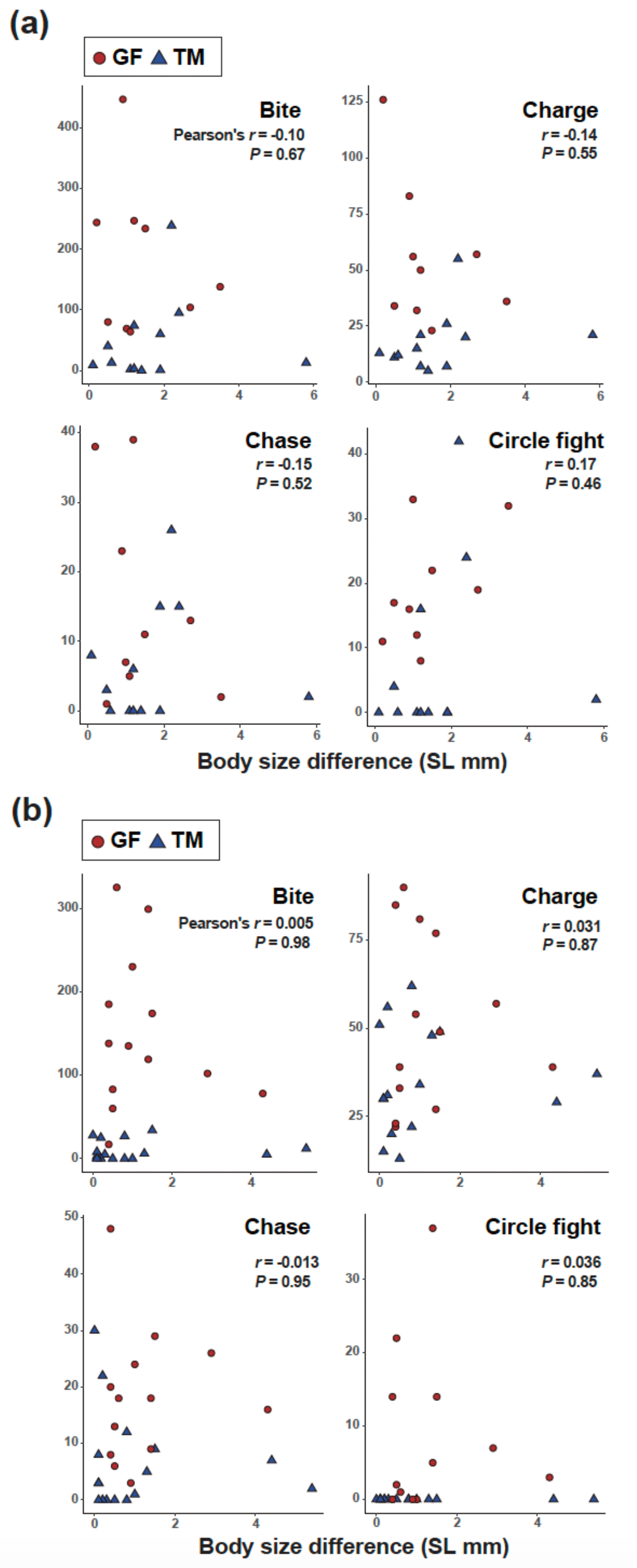

FIGURE S4 The relationship between the body size difference between two combating males and aggression-related behavioral components (the numbers of bites, charges, chases, and circle fights) in the one-on-one arena experiment using each GF and TM population. Results using (a) wild-caught and (b) common-garden males were exhibited (red circles: GF experiments; blue triangles: TM experiments), respectively. In each analysis, Pearson's correlation coefficient was shown with the *P*-value.

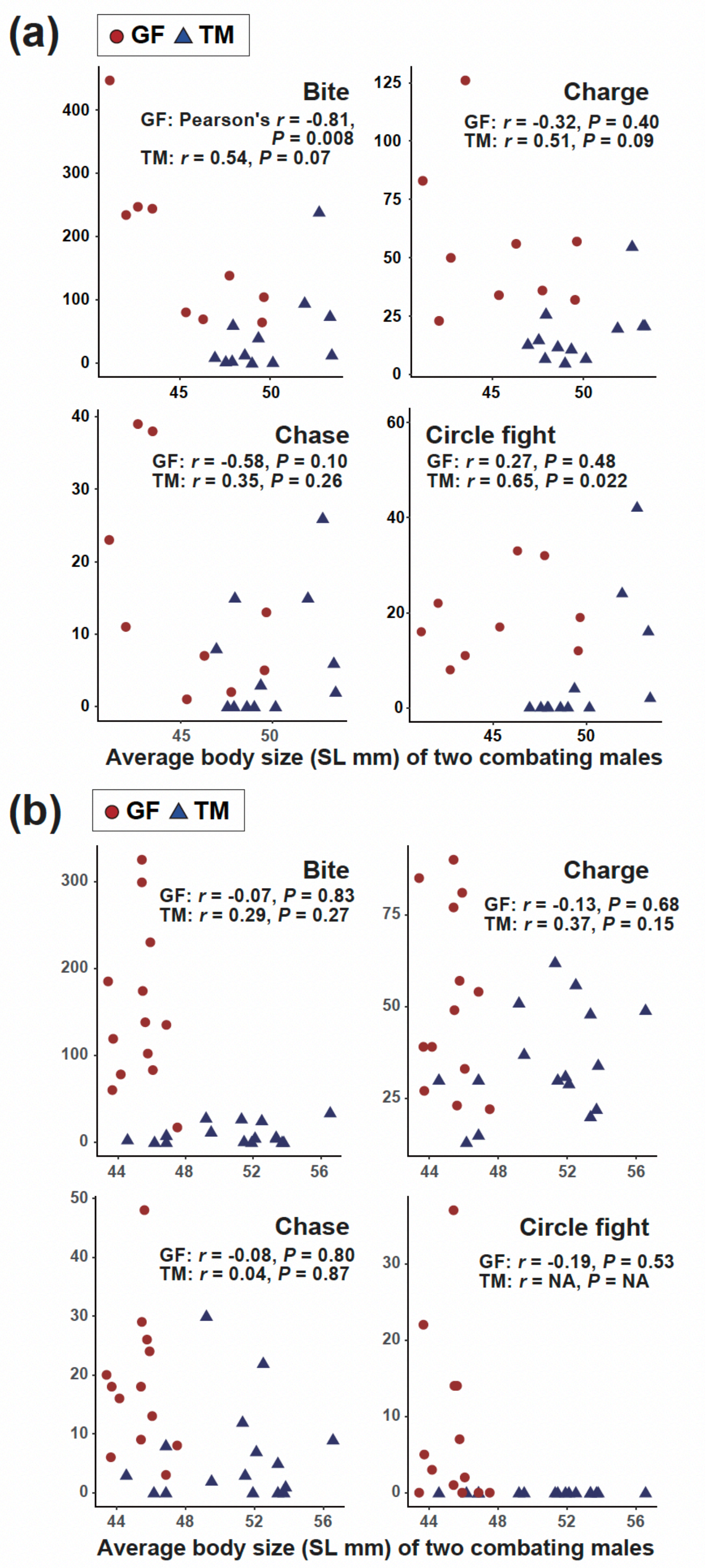

FIGURE S5 The relationship between the average body size of two combating males and aggression-related behavioral components (the numbers of bites, charges, chases, and circle fights) in the one-on-one arena experiment using each GF and TM population. Results using (a) wild-caught and (b) common-garden males were exhibited (red circles: GF experiments; blue triangles: TM experiments), respectively. In each analysis, Pearson's correlation coefficient was shown with the *P*-value.

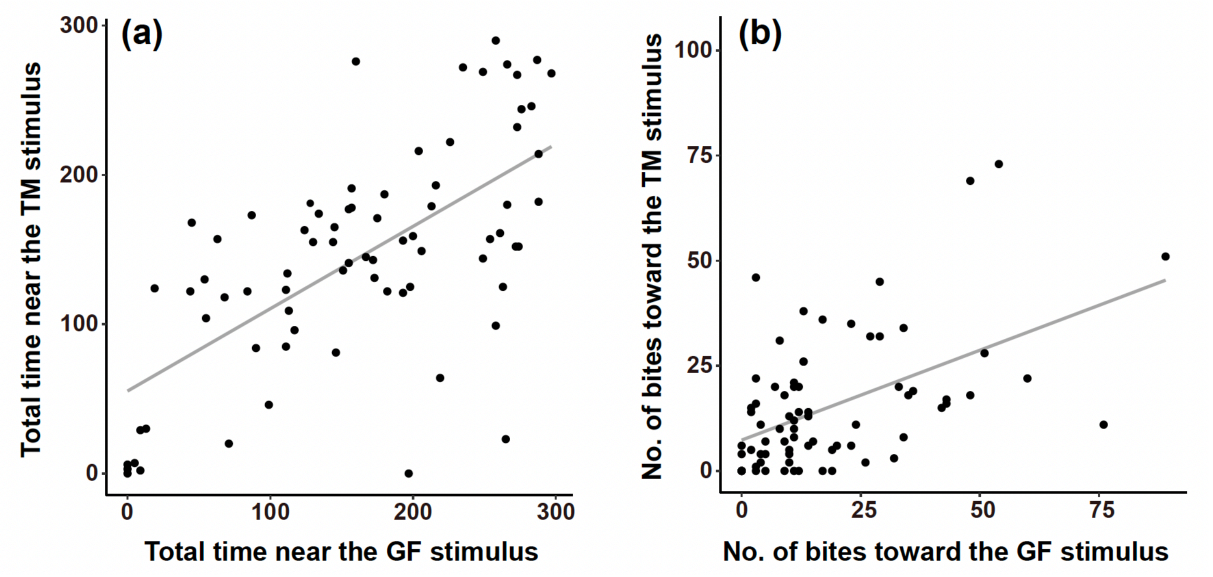

FIGURE S6 Correlation analysis of aggression-related behavioral components in F_2 males between the GF and TM stimulus males. (a) the total time (seconds) spent near the stimulus and (b) the number of bites toward the stimulus.

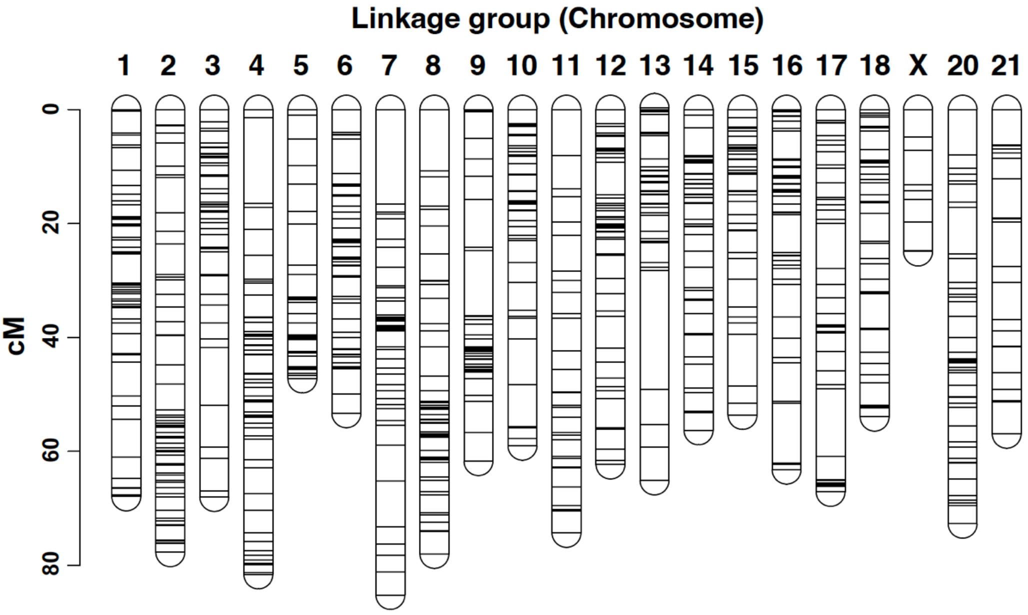

FIGURE S7 The linkage map constructed based on 980 ddRAD-SNP markers. The horizontal bars on each linkage group represent mapped SNP markers. Sex chromosome 19 was treated as an X chromosome (see text).

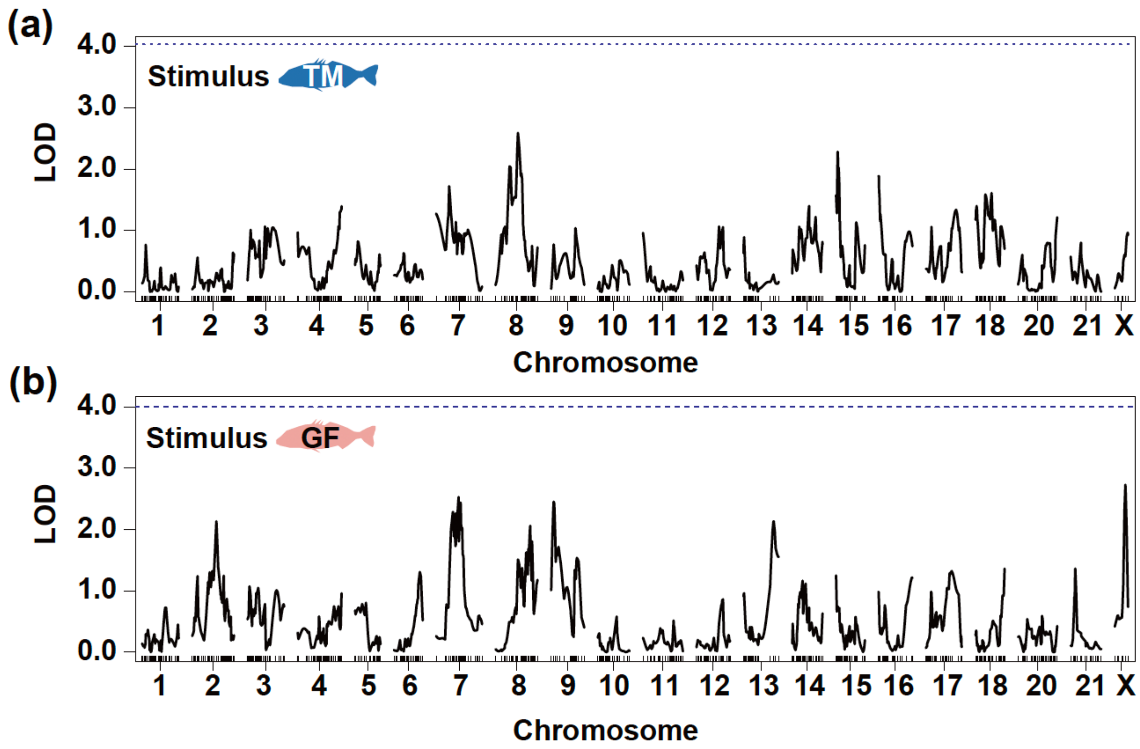

FIGURE S8 LOD score curve for the QTL analysis of an aggression-related behavioral component, i.e., the total time (seconds) spent near the (a) TM or (b) GF stimulus. A dotted horizontal line shows the significance level (95%). Sex chromosome 19 was treated as an X chromosome (see text).

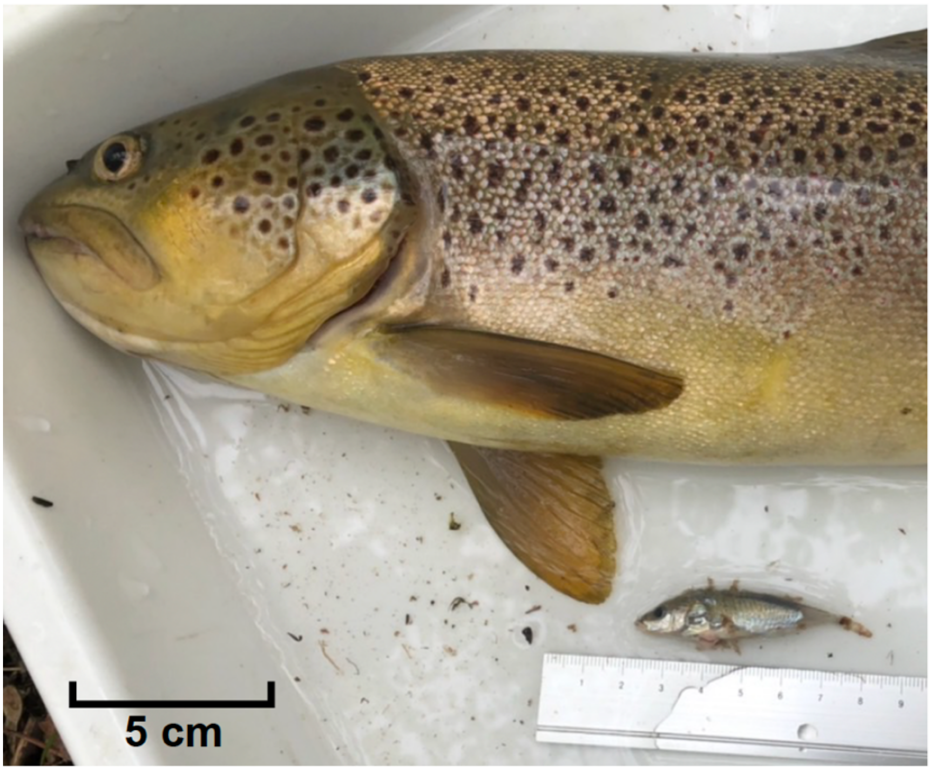

FIGURE S9 A photograph of a large brown trout and the stomach contents obtained by inserting the stomach pump into the trout. The trout's predation on adult (male) threespine stickleback was confirmed.
